# Diagnostic challenges in *Mycobacterium abscessus* pulmonary disease, particularly in patients with cystic fibrosis: toward an integrated precision framework

**DOI:** 10.1128/jcm.00812-26

**Published:** 2026-09-25

**Authors:** Vincent Le Moigne, Anne-Laure Roux, Clémence Beauruelle, Geneviève Héry-Arnaud, Jean-Louis Herrmann

**Affiliations:** 1 Université Paris-Saclay, UVSQ, Inserm, Infection et inflammation 569189 , Montigny-le-Bretonneux, France; 2 Assistance Publique-Hôpitaux de Paris. GHU Paris-Saclay, Service de Microbiologie, Hopital Raymond-Poincare 55531, Garches, France; 3 Center of Biome Analysis and Microbiota (CBAM), Brest University Hospital 26990 , Brest, France; 4 UnivBrest, Inserm, EFS, UMR 1078, GGB, Brest University 27002, Brest, France; 5 Department of Infectious Agents, Brest University Hospital 26990 , Brest, France; Medical College of Wisconsin, Milwaukee, Wisconsin, USA

**Keywords:** cystic fibrosis, *Mycobacterium abscessus*, diagnosis

## Abstract

*Mycobacterium abscessus* has emerged as one of the most formidable pathogens affecting people with cystic fibrosis (pwCF), driven by intrinsic antimicrobial resistance, complex taxonomy, and substantial diagnostic uncertainty. Despite major technological advances, establishing a diagnosis of pulmonary disease remains challenging, as traditional culture-based workflows are constrained by limited sensitivity, prolonged turnaround times. However, recent progress in proteomic and genomic technologies has markedly improved organism detection and taxonomic resolution. This review examines the contemporary diagnostic architecture for *M. abscessus* pulmonary infection in pwCF, identifying persistent gaps, and proposes a precision-oriented framework in which culture, proteomics, genomics, and host-response biomarkers converge to support earlier and more individualized clinical decision-making.

## INTRODUCTION

**Fig 1:**
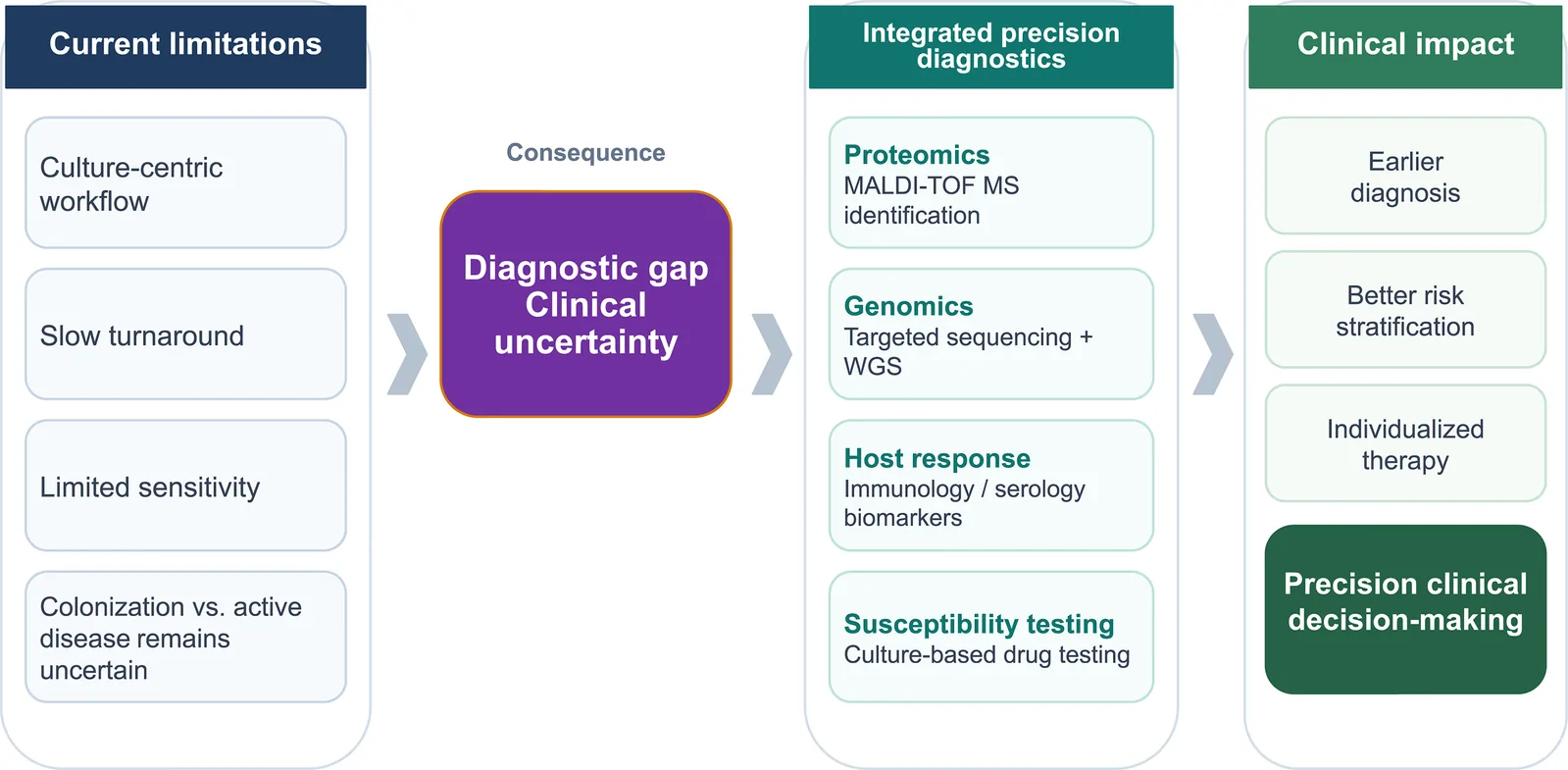
Closing the diagnostic gap in *M. abscessus* diagnosis, from culture-centric uncertainty to integrated precision diagnostics and actionable clinical decisions. Culture-based diagnosis remains slow, insensitive, and often ambiguous about colonization versus active disease. A multimodal precision workflow can close this gap and support earlier, more individualized treatment decisions.

*Mycobacterium abscessus* has emerged as one of the most clinically challenging pathogens in cystic fibrosis (CF), combining intrinsic multidrug resistance with complex taxonomy and substantial diagnostic uncertainty (1). Once considered a relatively rare opportunist, it is now recognized as a major threat to pulmonary health in people with CF (pwCF), where delayed or inaccurate diagnosis can directly influence therapeutic outcomes and transplant eligibility (2–4). Yet, despite significant technological progress, establishing a diagnosis of clinically relevant infection remains difficult. Conventional workflows—largely anchored in culture—struggle to reconcile analytical sensitivity with operational timeliness, while the expanding availability of proteomic, genomic, and host-response tools is reshaping the boundaries of diagnostic inference (Fig. 1).

*M. abscessus sensu lato* encompasses three subspecies—*M. abscessus* subsp. *abscessus*, *M. abscessus* subsp. *massiliense*, and *M. abscessus* subsp. *bolletii*—whose close genomic proximity belies clinically meaningful differences in antimicrobial susceptibility (5). Consequently, diagnostic precision is no longer a purely taxonomic objective but a prerequisite for therapeutic accuracy (4, 6).

Rather than reviewing diagnostic modalities in isolation, this article proposes an integrated precision framework in which microbiological, molecular, and immunological signals converge to support clinically actionable interpretation.

## ISOLATION OF *M*. *ABSCESSUS* FROM SPUTUM SAMPLES AND MAJOR SHORTCOMINGS

In routine microbiology practice, the diagnosis of non-tuberculous mycobacterial (NTM) pulmonary infections remains technically and clinically challenging (7), particularly in pwCF. Culture remains the reference standard for the isolation of *M. abscessus*. The most commonly used media include solid Löwenstein-Jensen and Middlebrook 7H10/7H11 (3), as well as the liquid Mycobacterial Growth Indicator Tube (MGIT; Becton Dickinson) system, which is based on Middlebrook 7H9 broth. Supplemented with OADC enrichment and the PANTA antibiotic mixture, MGIT is one of the most widely used liquid culture systems for the isolation and cultivation of mycobacteria.

Contamination remains a central determinant of culture performance and directly shapes the probability of recovering viable *M. abscessus* from respiratory specimens. In CF sputum, the high burden of rapidly growing bacteria—particularly *Pseudomonas aeruginosa*—creates a competitive environment in which even minimal residual contamination can rapidly overgrow culture media, rendering plates unreadable and masking the presence of mycobacteria. This phenomenon is especially problematic for *M. abscessus*, whose growth, although categorized as “rapid” among mycobacteria, remains substantially slower than that of common bacterial co-pathogens. As a result, incomplete decontamination leads to false-negative cultures not because of absence of the organism, but due to analytical failure driven by microbial overgrowth (7, 8). Conversely, increasing the intensity of decontamination to control contamination introduces a counterproductive effect by reducing the number of viable mycobacteria available for culture. Chemical agents such as sodium hydroxide exert a non-specific bactericidal effect that disproportionately impacts rapidly growing mycobacteria, which are more susceptible to chemical stress than slow-growing species. The cumulative effect of sequential or prolonged decontamination steps—such as the addition of oxalic acid targeting *Pseudomonas aeruginosa—*can markedly reduce viable inoculum size, in some cases below the threshold required for successful culture detection (7, 8). This creates a critical trade-off between contamination control and organism recovery, where optimizing one parameter inherently compromises the other. Importantly, the impact of these processing steps is not uniform and may depend on initial bacterial load, sample quality, and prior antibiotic exposure. Low-density *M. abscessus* populations, which are common in early or intermittent colonization, are particularly vulnerable to loss during processing, further contributing to underdiagnosis. In this context, culture negativity should be interpreted cautiously, especially in patients with compatible clinical and radiological features, as it may reflect methodological limitations rather than true absence of infection. Taken together, these observations highlight that pre-analytical processing is a major source of variability in *M. abscessus* detection. The balance between effective decontamination and preservation of mycobacterial viability remains a key unresolved challenge in routine microbiology workflows and provides a strong rationale for the development of alternative approaches that reduce reliance on harsh chemical processing (7, 8).

To mitigate contamination, selective agar media have been increasingly recommended as an alternative or complement to chemical decontamination strategies. *Burkholderia cepacia* selective agar (BCSA) was among the first to be repurposed for the isolation of rapidly growing mycobacteria (RGM), including *M. abscessus*, offering a practical compromise between contamination control and organism recovery (9, 10). Subsequent evaluations demonstrated that RGM-specific media can significantly improve recovery rates compared with conventional solid media such as Middlebrook or Löwenstein–Jensen, particularly in polymicrobial samples from patients with cystic fibrosis (9, 11, 12). Over the past decade, several novel or optimized culture media have been developed to further enhance the detection of NTM. These include chromogenic and highly selective formulations designed to suppress fast-growing bacterial and fungal contaminants while preserving mycobacterial viability (9, 13–16). Studies evaluating these media have consistently highlighted two key performance metrics: improved sensitivity for NTM recovery and reduced rates of culture overgrowth. However, performance varies depending on sample type, microbial burden, and laboratory workflow, and no single medium has emerged as universally optimal. More recently, NTM Elite Agar (bioMérieux) has been introduced as a next-generation selective medium specifically designed for the isolation of NTM from respiratory and environmental samples (17–19). A distinctive feature of this medium is its reported ability to support direct inoculation of clinical specimens without prior chemical decontamination (20). By eliminating sodium hydroxide or oxalic acid treatment, NTM Elite Agar preserves a greater proportion of viable mycobacteria, which may be particularly advantageous for fragile RGM populations such as *M. abscessus*. Multicenter evaluations have suggested improved recovery rates compared with conventional workflows, alongside effective inhibition of common contaminants, resulting in fewer uninterpretable cultures (17, 20). However, NTM Elite Agar has two important limitations: it is more expensive than conventional microbiological and mycobacterial media and is not suitable for the isolation of *Mycobacterium tuberculosis* complex. Other recent studies comparing RGM medium, BCSA, and automated liquid culture systems (e.g., MGIT) indicate that selective solid media may outperform liquid systems in heavily contaminated CF samples, where overgrowth can compromise detection despite faster theoretical growth kinetics (14). Conversely, liquid culture retains advantages in samples with low bacterial load, highlighting the complementary nature of these approaches. Taken together, these developments reflect a broader paradigm shift toward minimizing pre-analytical sample manipulation while relying on intrinsic media selectivity to control contamination. This strategy directly addresses one of the major limitations of traditional workflows—the loss of viable organisms during decontamination—while maintaining acceptable contamination rates. Nevertheless, important limitations remain, including variability in performance across laboratories, limited standardization, and the need for cost-effectiveness analyses. As a result, selective media such as BCSA, RGM formulations, and NTM Elite Agar are best viewed as components of an integrated diagnostic strategy, tailored to local epidemiology and laboratory constraints, rather than as standalone replacements for conventional culture methods.

## IDENTIFICATION METHODS AND DIFFICULTIES IN IDENTIFYING *M*. *ABSCESSUS*

Identification of *M. abscessus* presents several challenges and has evolved substantially over recent decades, driven by the limitations of phenotypic methods and the advances in molecular tools. *M. abscessus* was first isolated in 1952–1953 (21) and was originally considered a subspecies of *Mycobacterium chelonae* owing to close morphological and biochemical similarities and similarity at the 16S rRNA level (22). Both were grouped among RGM. In 1972, they were divided into two subspecies (22). With advances in DNA-DNA hybridization and later multilocus sequence analysis, clear genetic differences emerged. In 1992, *M. abscessus* was officially separated from *M. chelonae* and designated as its own species (23). This taxonomic revision was further supported by differences in clinical presentation and antimicrobial susceptibility, particularly inducible macrolide resistance in *M. abscessus* (4).

Accurate identification of RGM has evolved from morphology-based inference to multilayered genomic interpretation. This transition reflects a broader shift in clinical microbiology: diagnostic precision is pursued not for taxonomic completeness alone but as a prerequisite for therapeutic accuracy. Nowhere is this more evident than within the *M. abscessus* complex (MABC), whose members (*Mycobacterium abscessus* subsp. *abscessus*, *Mycobacterium abscessus* subsp. *massiliense*, and *Mycobacterium abscessus* subsp. *bolletii*) combine extreme genetic proximity with clinically meaningful differences in antimicrobial susceptibility.

Phenotypic methods retain operational value as an initial classification step. Rapid colony formation within 3-7 days on Lowenstein-Jensen and Middlebrook media reliably separates RGM from slow-growing species such as *M. tuberculosis* or other NTM as *Mycobacterium avium*. Colonies are typically non-pigmented, cream-white, and morphologically smooth or rough—features that support group-level recognition but lack specificity. Biochemical testing further consolidates this preliminary assignment. A positive 3-day arylsulfatase reaction, catalase activity, exuberant urease production, and tolerance to 5% NaCl align with established RGM metabolic signatures. Conversely, negative niacin production, absent nitrate reductase activity, and variable Tween 80 hydrolysis contribute little discriminatory value (24). The limitation is structural rather than technical: phenotypic convergence within the MABC precludes reliable subspecies differentiation. Accordingly, phenotype should be understood as a probabilistic filter that accelerates downstream testing.

Beyond its role as a rapid identification tool, the analytical performance of matrix-assisted laser desorption/ionization time-of-flight mass spectrometry (MALDI-TOF MS) for *M. abscessus* warrants a more nuanced interpretation, particularly regarding subspecies discrimination. At the complex level, MALDI-TOF demonstrates high accuracy, with most studies reporting reliable identification of the *M. abscessus* species, from solid as well as liquid media when appropriate extraction protocols and updated spectral databases are used (25–27) (Table 1). This performance has made it a cornerstone of first-line identification in clinical microbiology laboratories, significantly reducing turnaround time compared with conventional phenotypic methods. However, discrimination at the subspecies level (i.e., *M. abscessus* subsp. *abscessus*, *massiliense*, and *bolletii*) remains a major limitation. The proteomic similarity between these subspecies results in highly overlapping spectral profiles, limiting the ability of standard algorithms to achieve consistent differentiation. Reported accuracy for subspecies identification using conventional MALDI-TOF workflows is variable and generally insufficient for clinical decision-making, with frequent misclassification between subsp. *abscessus* and subsp. *massiliense* (25, 28). This limitation is primarily biological rather than technical, reflecting the high genomic and proteomic conservation within these species. Recent advances have explored the use of enhanced bioinformatic approaches, including machine learning–based classification of MALDI-TOF spectra, to improve subspecies resolution. These methods have demonstrated promising results in controlled settings, with improved discrimination accuracy compared with conventional database matching (28–30). However, their implementation in routine diagnostics remains limited by the need for large, well-curated spectral data sets, standardization across platforms, and validation in multicenter studies. As such, these approaches should currently be considered investigational rather than ready for widespread clinical adoption. Importantly, several pre-analytical and technical factors influence MALDI-TOF performance, including culture conditions (solid versus liquid media), extraction protocols, and database completeness. Suboptimal extraction, particularly for mycobacteria with lipid-rich cell walls, can significantly reduce identification accuracy. Moreover, database limitations remain a critical bottleneck, as incomplete or poorly annotated reference spectra may impair both species- and subspecies-level identification. In practical terms, MALDI-TOF MS should therefore be regarded as a rapid and reliable method for identification of the *M. abscessus* species, but not as a standalone tool for subspecies discrimination. Given the clinical importance of distinguishing subspecies—particularly in relation to inducible macrolide resistance—confirmation by molecular methods remains essential. This reinforces the role of MALDI-TOF within a layered diagnostic strategy, where rapid proteomic identification is complemented by targeted sequencing to achieve clinically actionable resolution.

**TABLE 1:** Key capabilities and limitations of MALDI-TOF for rapidly growing mycobacteria

Feature	Performance
Identification of *M. abscessus* species	Reliable
Subspecies discrimination (*abscessus, massiliense, bolletii*)	Limited/unreliable
Identification of other RGM (*M. fortuitum* complex, *M. chelonae* complex)	High accuracy (at complex level)
Turnaround time	Very rapid on liquid or solid culture (28–30)
Extraction requirement	Recommended (formic acid-acetonitrile)
Role in workflow	First-line identification; molecular confirmation advised

The taxonomic clarity now associated with the MABC is largely the product of molecular inquiry. Early sequencing of conserved loci—including 16S rRNA, hsp65, rpoB, and the 16S-23S internal transcribed spacer—first exposed the genetic heterogeneity masked by phenotypic similarity (31–34). Subsequent work demonstrated that single-target sequencing could not consistently resolve these taxa, prompting a shift toward multilocus strategies (32–34).

A decisive functional marker emerged with characterization of *erm(41)* (35), whose structural integrity governs inducible macrolide resistance. Because the distribution of functional versus truncated variants differs among subspecies, *erm*(41) sequencing simultaneously informs taxonomy and therapeutic prediction (36). Whole-genome sequencing (WGS) provides the highest-resolution framework, capturing subtle phylogenetic gradients that define intra-complex diversification (37–40).

In conclusion, while culture and phenotypic testing remain indispensable for isolating *M. abscessus*, their limited resolution and prolonged turnaround constrain their standalone diagnostic value. Proteomic technologies, such as MALDI-TOF, markedly accelerate complex-level identification but do not obviate the need for molecular analysis. Rather, optimal diagnostic accuracy emerges from a layered strategy in which rapid proteomic classification is coupled with targeted sequencing to achieve subspecies resolution and predict clinically consequential resistance mechanisms, particularly inducible macrolide resistance.

## ANTIMICROBIAL SUSCEPTIBILITY PROFILE

Antimicrobial susceptibility testing provides clinically informative phenotypic signals for distinguishing RGM, particularly *M. abscessus*, *Mycobacterium chelonae*, and *Mycobacterium fortuitum*. Macrolide susceptibility, especially to clarithromycin, represents the most clinically consequential discriminator. *M. abscessus* may exhibit inducible macrolide resistance mediated by a functional *erm*(41) gene (35), typically becoming apparent only after extended incubation (up to 14 days) (41). Failure to detect delayed resistance risks inappropriate regimen selection. In contrast, *M. chelonae* lacks a functional *erm*(41) gene and is usually clarithromycin susceptible (42). However, it is worth noting that the plasmid-encoded inducible macrolide resistance gene *erm*(55)^P^ has recently been identified in *M. chelonae* and other rapidly growing mycobacteria (RGM) (43).

Tobramycin susceptibility constitutes a second robust discriminator: *M. abscessus* is resistant in most isolates, whereas *M. chelonae* is often susceptible. Broader patterns further support species-level distinction: both *M. abscessus* and *M. chelonae* commonly exhibit resistance to fluoroquinolones, whereas *M. fortuitum* is often susceptible. Imipenem and cefoxitin show variable activity against *M. abscessus*, limited activity against *M. chelonae*, and more reliable activity against *M. fortuitum*. Amikacin generally retains activity across these species and remains central to severe RGM disease management (Table 2) (4). These susceptibility profiles represent general trends and should not replace isolate-specific antimicrobial susceptibility testing.

**TABLE 2:** Typical antimicrobial susceptibility profiles of the major rapidly growing mycobacteria (RGM)

Antibiotic	*M. abscessus* complex			*M*. *chelonae*	*M*. *fortuitum*
	Subsp. *abscessus*	Subsp. *massiliense*	Subsp. *bolletii*		
Clarithromycin^*a*^	Usually inducible resistance [functional *erm(41)*]	Usually susceptible [non-functional *erm(41)*]	Usually inducible resistance [functional *erm(41)*]	Susceptible	Usually susceptible
Tobramycin	Poor activity	Poor activity	Poor activity	Susceptible	Poor activity
Fluoroquinolones	Poor activity	Poor activity	Poor activity	Poor activity	Susceptible
Cefoxitin	Variable susceptibility	Variable susceptibility	Variable susceptibility	Resistant	Susceptible
Imipenem	Variable susceptibility	Variable susceptibility	Variable susceptibility	Resistant	Susceptible
Amikacin^*a*^	Susceptible	Susceptible	Susceptible	Susceptible	Susceptible

^*a*^ In France, the antimicrobial susceptibility committee of the French Microbiology Society propose to evaluate those two antibiotics in addition to tigecycline (44).

In addition to species-specific susceptibility patterns, the methodological framework used for antimicrobial susceptibility testing (AST) also critically influences result interpretation. Phenotypic AST for *M. abscessus* relies primarily on broth microdilution methods, which are currently recommended as the reference standard by the Clinical and Laboratory Standards Institute (CLSI) and the European Committee on Antimicrobial Susceptibility Testing (EUCAST) guidances (41, 44). This approach enables determination of minimum inhibitory concentrations (MICs) across a standardized panel of antimicrobial agents and allows detection of clinically relevant resistance phenotypes, including inducible macrolide resistance. A key strength of broth microdilution lies in its reproducibility and its ability to capture the integrated effect of multiple resistance mechanisms, including those not predictable from genotype alone (4, 41). However, this method is technically demanding and associated with several limitations. Interpretation of MICs requires experienced personnel, as visual reading may be complicated by trailing growth, clumping, or heterogeneous populations. In addition, prolonged incubation—up to 14 days—is necessary to reveal inducible resistance mediated by *erm*(41), particularly for clarithromycin, which delays result availability and may complicate clinical decision-making (41). Furthermore, clinical breakpoints are not established for all antimicrobial agents, and the correlation between *in vitro* MIC values and *in vivo* efficacy remains imperfect for several drug classes. Alternative phenotypic methods, such as agar dilution and gradient diffusion techniques (e.g., Etest), have been evaluated but are less standardized for RGM and may yield variable results depending on media composition, inoculum size, and incubation conditions. Similarly, automated susceptibility testing systems widely used in routine bacteriology are not validated for *M. abscessus*, limiting scalability and inter-laboratory comparability. Liquid culture-based systems may theoretically offer faster growth detection, but their use for AST remains limited and not fully standardized for RGM. Importantly, all phenotypic approaches are influenced by pre-analytical factors such as inoculum density and prior antibiotic exposure, which may affect reproducibility. Despite these challenges, phenotypic AST remains indispensable in the diagnostic workflow, as it provides direct functional evidence of antimicrobial susceptibility. Molecular assays can complement this approach by rapidly identifying specific resistance determinants, but they do not capture the full complexity of resistance mechanisms. Consequently, optimal practice relies on a combined strategy in which phenotypic testing is used as the reference method, supported by targeted molecular assays to accelerate detection of key resistance markers.

## MOLECULAR METHODS FOR *M*. *ABSCESSUS* RESPIRATORY INFECTIONS

The evolution of identification strategies highlights that diagnostic certainty in NTM disease increasingly depends on analytical depth rather than methodological singularity. Even refined culture-based workflows remain constrained by prolonged turnaround times and imperfect sensitivity. These limitations are particularly consequential in respiratory medicine, where the central diagnostic dilemma is not merely detection but attribution—distinguishing active infection from intermittent colonization. This distinction is particularly critical in CF, where intermittent or low-level detection may reflect environmental acquisition or transient airway carriage rather than progressive pulmonary disease. Future diagnostic frameworks will therefore need to integrate microbiological persistence, quantitative burden, host immune signatures, and radiological evolution to better discriminate colonization from true disease and support therapeutic decision-making.

Because environmental exposure to NTM is common, a single positive sputum specimen rarely establishes pathogenicity. Diagnostic confidence instead emerges from microbiological persistence, justifying repeated respiratory sampling as a foundational principle. Within this context, adjunctive tools such as serological assays and blood-based metagenomic next-generation sequencing (mNGS) are gaining attention for their potential to reinforce diagnostic probability when clinical and radiographic signals remain ambiguous.

Molecular assays now function as the primary culture-independent backbone for early detection. A major conceptual shift in the field is the emergence of direct-from-specimen diagnostics, which aim to bypass the intrinsic limitations of culture-based workflows. By enabling pathogen detection, subspecies identification, and resistance profiling directly from sputum, these approaches may substantially reduce diagnostic latency and preserve analytical sensitivity in low-burden or intermittently colonized patients. Although still limited by host DNA background, polymicrobial complexity, and cost, direct sequencing strategies—including targeted enrichment and metagenomic workflows—represent one of the most promising developments for future *M. abscessus* diagnostics.

Sequencing of conserved targets—including *rpo*B and *hsp*65—and multilocus sequence typing (MLST) provides reliable phylogenetic positioning while shortening time to identification (45). Line probe assays permit rapid, sometimes direct, detection from respiratory specimens, but their probe-dependent architecture inherently limits genomic breadth and can leave taxonomic blind spots (46–49).

NGS is particularly valuable for the detection of resistance determinants in *M. abscessus*, a species characterized by complex and multifactorial antimicrobial resistance. Beyond simple mutation screening, sequencing provides a comprehensive view of both known and emerging resistance mechanisms, enabling more precise therapeutic guidance. Key targets include mutations in the *rrl* gene associated with acquired macrolide resistance, the *erm(41)* gene responsible for inducible macrolide resistance, and mutations in the *rrs* gene linked to amikacin resistance (35–37). In addition, sequencing approaches can identify less common or newly described resistance-associated variants, as well as mutations in genes involved in drug targets, efflux systems, or regulatory pathways, which may contribute to phenotypic resistance but are not captured by routine molecular assays (37–40). Compared with targeted molecular tests, sequencing offers the advantage of multiplex detection, allowing simultaneous identification of species, subspecies, and resistance determinants within a single workflow. This is particularly relevant for *M. abscessus*, where taxonomic resolution and resistance prediction are closely linked, especially in the context of macrolide susceptibility. Whole-genome sequencing (WGS) further enhances this capability by providing high-resolution genomic data that can inform both clinical management and epidemiological investigations, including transmission analysis in CF populations (38–40). However, the performance of sequencing approaches varies depending on the method used and the type of sample analyzed. Sanger sequencing remains widely used in routine practice for identification from positive cultures, typically targeting conserved genes such as *rpoB*, *hsp65*, and 16S *rRNA*. When applied to cultured isolates, Sanger sequencing provides high analytical accuracy at the species level and, when combined in multilocus approaches, can achieve reliable subspecies discrimination (31–34). Its main limitations include dependence on prior culture, limited throughput, and restricted ability to detect mixed populations or minority variants. In contrast, NGS technologies offer substantially higher resolution and broader analytical scope. When applied to cultured isolates, targeted NGS panels or WGS enable accurate subspecies identification, comprehensive detection of resistance-associated mutations, and improved characterization of strain diversity (37–40, 50). These approaches also allow detection of heteroresistance and minor subpopulations that may be clinically relevant but remain undetected by conventional methods. Direct sequencing from clinical specimens, particularly sputum from patients with CF, represents a major advance but remains technically challenging. High human DNA content, low mycobacterial load, and complex polymicrobial backgrounds can reduce analytical sensitivity and require optimized sample preparation, including host DNA depletion and targeted enrichment strategies. Metagenomic NGS has demonstrated the ability to detect *M. abscessus* directly from respiratory samples, with variable sensitivity depending on sequencing depth and bacterial burden (51). While this approach offers the potential for earlier diagnosis and resistance profiling without prior culture, its routine implementation is currently limited by cost, turnaround time, and the need for specialized bioinformatics pipelines. Overall, Sanger sequencing and NGS should be considered complementary rather than competing approaches within a tiered diagnostic strategy. Sanger sequencing provides a robust and accessible method for identification from cultured isolates, whereas NGS delivers enhanced resolution, multiplex resistance detection, and the possibility of direct-from-sample analysis. Together, these technologies contribute to a more rapid and precise characterization of *M. abscessus*, supporting both clinical decision-making and epidemiological surveillance. In addition, NTMs must be considered within their respiratory ecological context and, studying the microbiome can help us better understand the transition from presence/colonization to clinically significant lung disease (52).

More recently, bacterial detection has been achieved by targeting their degraded forms directly in circulating blood. Pathogens release cell-free DNA (cfDNA) into the host circulation during cell lysis and immune clearance. This cfDNA can be detected in blood, providing a minimally invasive approach for pathogen identification, including mycobacteria, that does not rely on sampling the primary site of infection (53). To improve the sensitivity of cfDNA detection, CRISPR-based approaches have been developed. Specifically, in cases of *Mycobacterium avium* complex (MAC) lung disease, cfDNA from MAC species has been detected in serum during active disease using a CRISPR-based assay, highlighting its potential utility for early and non-invasive diagnosis (54). More recently, CRISPR-based technologies have been combined with circulating cfDNA analysis, as exemplified by CADEM (CRISPR-Assisted Detection via Enrichment of *Mycobacterium*-derived cfDNA using Microfluidic technology) (55), which uses liquid biopsy samples, including serum, to detect mycobacterial cfDNA, including MABC, for the diagnosis of pulmonary disease.

These technologies should be viewed as complementary to rather than replacements for culture. Culture remains indispensable for phenotypic susceptibility testing and epidemiological surveillance; molecular and metagenomic tools instead compress diagnostic latency and refine early judgment. Overall, the field increasingly favors concurrency over sequence—rapid genomic insight guiding initial management while culture anchors functional validation.

## IMMUNOLOGICAL APPROACHES FOR THE DIAGNOSIS OF *M*. *ABSCESSUS* PULMONARY DISEASE

Immunological diagnosis—defined as the assessment of the host response to bacterial infection through serological antibody detection or characterization of T-lymphocyte responses—has long represented a diagnostic alternative for pathogens that are difficult to cultivate, non-cultivable, or require specialized conditions to prevent overgrowth by commensal flora, as described above for mycobacteria and *Legionella*. Historically, such strategies have proven valuable for infections caused by organisms such as *Borrelia burgdorferi*, *Treponema pallidum,* and *Francisella tularensis*.

In tuberculosis caused by members of the *M. tuberculosis* complex, however, these approaches—particularly serology—have largely failed to demonstrate sufficient diagnostic performance (56). Technological and commercial development instead focused on T-cell–based assays, notably interferon-γ release assays (IGRAs), introduced in part to compensate for the limitations of the tuberculin skin test. None of the commercially available serological tests have proven sufficiently accurate to replace smear microscopy (57, 58).

By contrast, the diagnostic challenges encountered in respiratory infections due to nontuberculous mycobacteria (NTM), including *M. abscessus*, differ substantially—particularly in patients with CF or bronchiectasis. A crucial factor is the marked disparity in disease incidence. Tuberculosis incidence in countries such as France remains approximately 6 per 100,000 inhabitants (59), whereas NTM infections in CF or bronchiectasis populations are reported as percentages, approaching 10% in recent French epidemiological data and ranging from 6% to 13%—with wider variability—in U.S. studies (60–63 and Héry-Arnaud, Le Moigne, and Herrmann, unpublished results). Such epidemiological differences profoundly modify the predictive values of diagnostic tests.

Recent therapeutic advances in CF further complicate direct microbiological diagnosis. The introduction of triple CFTR modulator therapy (64) has significantly improved respiratory symptoms but has concurrently reduced sputum frequency and volume. As current international guidelines require at least two positive respiratory samples to establish NTM pulmonary disease (65, 66), the feasibility of culture-based diagnosis is increasingly challenged by limited specimen availability. This raises critical questions regarding the monitoring of patients potentially colonized or infected with NTM, particularly as the impact of CFTR triple therapy on NTM epidemiology remains uncertain. Similarly, implementing the recommended annual screening for new NTM colonization or infection in pwCF may become increasingly complex (65).

Interpretation is further complicated by the nonspecific clinical presentation and radiological background of CF and bronchiectasis, where baseline abnormalities may overlap with manifestations of NTM disease. In this context, immunological tools—such as serology or T-cell-based assays—may offer an alternative means of identifying patients mounting an immune response suggestive of prior or ongoing infection.

Major serological investigations initially focused on detecting IgG and IgA antibodies directed against the *Mycobacterium avium* complex (MAC). These studies aimed to stratify patients according to antibody levels potentially reflecting disease activity, correlate serological findings with radiological features, or associate them with smear positivity within the preceding six months (67–71).

A longitudinal cohort of 305 pwCF, with a median of five NTM cultures per patient, demonstrated that the four individuals with MAC infection exhibited median antibody levels 22-fold higher than those without a history of infection (69). Although confirmation in larger cohorts was recommended, the authors proposed stratifying pwCF into high- and low-risk groups based on antibody titers, potentially guiding the frequency of microbiological surveillance (69).

Particular attention has been devoted to IgA antibodies targeting MAC glycopeptidolipid (GPL) core antigens (72–77). Numerous assays have been developed, with reported sensitivities ranging from 59% to 82% and specificities from 94% to 99% (68, 71, 78–80). It should be noted that GPL-based assays primarily detect antibodies directed against glycopeptidolipids expressed by smooth morphotypes. Nevertheless, morphotype switching from smooth to rough is a well-recognized feature of *M. abscessus* during disease progression, and mixed smooth and rough populations have also been reported in the *M. avium* complex. Therefore, patients are likely to encounter GPL antigens during at least part of the course of infection, which may contribute to the diagnostic performance of GPL-based serological assays.

In bronchiectasis populations, anti-GPL core IgA levels demonstrated remarkable accuracy for identifying NTM pulmonary disease (NTM-PD), with an area under the receiver operating characteristic curve of 0.886 (95% CI, 0.800−0.973), and effectively discriminated patients with NTM-PD from those with NTM isolation not meeting diagnostic criteria (AUC 0.816; 95% CI, 0.687−0.945) (77). IgA titers also decline following surgical intervention, supporting their role as markers of infection resolution, and correlate with antibiotic treatment efficacy (72). Accordingly, IgA detection may serve as a tool for predicting recurrence and monitoring disease activity.

Until recently, few studies specifically addressed serological characterization of *M. abscessus* pulmonary infection (81, 82). These investigations employed either the mycobacterial antigen A60 or whole-cell extracts, both targeting IgG responses. In the study by Qvist et al., more than 300 pwCF were included, and 3,480 samples were tested against an *M. abscessus* whole-cell extract, yielding sensitivity and specificity values of 95% and 73%, respectively. An algorithm was subsequently proposed to stratify patients into high- and low-risk groups for *M. abscessus* infection (82). The French study evaluated the A60 antigen (AndaTB), typically used for tuberculosis diagnosis, in a cohort of 184 pwCF aged 12 ± 5 years. Because patients with NTM infection often exhibit anti-A60 antibodies, the authors assessed its diagnostic value in individuals with and without *M. abscessus* infection. Depending on the age-adjusted cutoff selected, sensitivity and specificity reached 87% and 95%, respectively. Antibody levels were nearly fourfold higher in patients meeting ATS diagnostic criteria than in culture-positive individuals lacking compatible clinical and radiological findings, and sixfold higher than in NTM culture-positive patients who failed to meet the ATS diagnostic criteria for NTM pulmonary disease (81).

Specific clinical scenarios may therefore justify use of this assay—for example, in cases with a high index of suspicion to guide microbiological investigations, evaluate disease activity, or monitor treatment response. Its role in screening remains uncertain and should be considered in light of local epidemiology. Despite promising results, confirmation in larger cohorts is required.

A study assessing IgG responses to both phospholipase C (PLC) and a TLR2-positive *M. abscessus* surface extract (TLR2eF) (83) demonstrated notable diagnostic performance individually and in combination (84), achieving sensitivity of 93.9% and specificity of 80.7% for detecting culture-positive pwCF. In this context, the negative predictive value reached 99.7%, whereas the positive predictive value was 15.4%.

Detection of IgA directed against PLC and TLR2eF was likewise shown to be effective (79). Combining IgA and IgG responses further improved diagnostic metrics, particularly yielding a negative predictive value of 98.5% (85). These findings support the use of these antigens as valuable complementary tools for characterizing pwCF potentially infected with NTM, especially given the clinical importance of a confident exclusion of infection when culture-based methods are challenging.

Another study employing a total lysate of heat-killed *M. abscessus* as antigen evaluated both IgG and IgA responses (86), highlighting the potential diagnostic relevance of the IgG3 subclass. Synthetic mycolic acid antigens have also been explored: individual trehalose dimycolates combined with a GPL-core antigen effectively distinguished infected from non-infected individuals, achieving specificity of 88% and sensitivity up to 88%, which increased to 93% specificity when multiple antigens were combined (87).

More recently, a novel glycosylated lipo-octapeptide GPL, designated GL8P, was shown to be specifically recognized by pwCF infected with *M. abscessus* (88). Notably, the biosynthetic gene locus appears restricted to the *M. abscessus–chelonae* complex and a limited number of closely related phylogenetic species (*Mycobacterium immunogenum*, *Mycobacterium salmoniphilum*, and *Mycobacterium saopaulense*), while differing from predicted products in MAC species (*M. avium sensu stricto*, *Mycobacterium hominissuis*, *Mycobacterium intracellulare*, and *Mycobacterium chimaera*). Such specificity may represent an important step toward improved taxonomic precision in NTM serology.

## CONCLUSION

Diagnosing pulmonary *M. abscessus* infection in CF remains a multidimensional challenge, shaped by microbial complexity, specimen limitations, and rapidly evolving therapeutic landscapes. The persistence of surveillance gaps, the requirement for repeated sampling despite low culture positivity, and the difficulty in distinguishing colonization from progressive disease collectively underscore the limits of traditional diagnostic paradigms.

The future of *M. abscessus* diagnostics will depend less on the performance of individual technologies than on the coherence of the architecture that integrates them. Layered strategies combining culture, proteomics, genomics, and host-response biomarkers have the potential to transform fragmented microbiological data into clinically actionable knowledge. Within this framework, immunological assays—particularly those offering high negative predictive value—may prove pivotal by enabling confident disease exclusion, reducing unnecessary therapeutic exposure, and focusing intensive management on patients most likely to benefit.

Future diagnostic paradigms will likely integrate pathogen genomics with host-response signatures to generate predictive models capable of distinguishing transient colonization from incipient disease. Beyond pathogen- and immune-targeted assays, host-derived biomarkers, including transcriptomic signatures that have shown considerable promise in tuberculosis (89), may also contribute to future precision diagnostic strategies for nontuberculous mycobacterial disease, although their application to *M. abscessus* infection remains at an early stage. Advances in machine learning applied to multimodal microbiological data sets may further enable risk stratification and individualized therapeutic decision-making, marking a decisive shift toward precision medicine in the field of infectious disease (Fig. 1).

As the burden of NTM disease continues to rise, diagnostic excellence must evolve from organism detection toward probabilistic, precision-oriented interpretation. The ability to synthesize multidimensional evidence into decisive clinical insight will increasingly define the new generation standard of care in CF microbiology.
